# Sensitivity Evaluation of Enveloped and Non-enveloped Viruses to Ethanol Using Machine Learning: A Systematic Review

**DOI:** 10.1007/s12560-023-09571-2

**Published:** 2023-12-05

**Authors:** Aken Puti Wanguyun, Wakana Oishi, Daisuke Sano

**Affiliations:** 1 Department of Frontier Science for Advanced Environment, Graduate School of Environmental Studies, Tohoku University, Sendai, Japan; 2 Department of Civil and Environmental Engineering, Graduate School of Engineering, Tohoku University, Sendai, Japan

**Keywords:** Ethanol, Inactivation, Enveloped viruses, Non-enveloped viruses, Decision tree, Random forest

## Abstract

Viral diseases are a severe public health issue worldwide. During the coronavirus pandemic, the use of alcohol-based sanitizers was recommended by WHO. Enveloped viruses are sensitive to ethanol, whereas non-enveloped viruses are considerably less sensitive. However, no quantitative analysis has been conducted to determine virus ethanol sensitivity and the important variables influencing the inactivation of viruses to ethanol. This study aimed to determine viruses’ sensitivity to ethanol and the most important variables influencing the inactivation of viruses exposed to ethanol based on machine learning. We examined 37 peer-reviewed articles through a systematic search. Quantitative analysis was employed using a decision tree and random forest algorithms. Based on the decision tree, enveloped viruses required around ≥ 35% ethanol with an average contact time of at least 1 min, which reduced the average viral load by 4 log_10_. In non-enveloped viruses with and without organic matter, ≥ 77.50% and ≥ 65% ethanol with an extended contact time of ≥ 2 min were required for a 4 log_10_ viral reduction, respectively. Important variables were assessed using a random forest based on the percentage increases in mean square error (%IncMSE) and node purity (%IncNodePurity). Ethanol concentration was a more important variable with a higher %IncMSE and %IncNodePurity than contact time for the inactivation of enveloped and non-enveloped viruses with the available organic matter. Because specific guidelines for virus inactivation by ethanol are lacking, data analysis using machine learning is essential to gain insight from certain datasets. We provide new knowledge for determining guideline values related to the selection of ethanol concentration and contact time that effectively inactivate viruses.

**Supplementary Information:**

The online version contains supplementary material available at 10.1007/s12560-023-09571-2.

## Introduction

The emergence of viral infectious diseases poses a severe threat to the human population worldwide, given the associated high morbidity and mortality. According to the World Health Organization (WHO), in 2019, lower respiratory tract infections and gastroenteritis infections were the most common viral infectious diseases, resulting in deaths across the world (Department of Data & Analytics, 2020). The recent global outbreak of the coronavirus disease (COVID-19), caused by infection with severe acute respiratory syndrome coronavirus 2 (SARS-CoV-2), has caused more than five million deaths globally (Adam, 2022). Severe gastroenteritis infections have also become the primary cause of mortality globally in children under 5 years of age, leading to the death of more than 400,000 children per year (Troeger et al., 2017).

Viruses can be transmitted through person-to-person contact, airborne transmission, or contact with contaminated objects or surfaces, referred to as fomites (Kraay et al., 2018; Kutter et al., 2018; van Seventer & Hochberg, 2016). Several types of viruses can also be transmitted through contaminated water and food, causing water- and food-borne infections (Lopman et al., 2012; Rzezutka & Cook, 2004). One effective intervention strategy for fomite transmission is using disinfectants, such as alcohol-based solutions (Castañ et al., 2021). During the COVID-19 pandemic, WHO recommended improving daily hygiene practices, such as regular handwashing and the use of alcohol-based sanitizers (Ghafoor et al., 2021).

Ethanol is preferably used as a disinfectant to inactivate bacteria and enveloped viruses, as it effectively dissolves lipid membranes (Ali et al., 2021). Ethanol can interfere with the structure of the viral lipid envelope, causing disassembly of the virus particle structure, including detachment of the spike protein from the lipid bilayer (Sato et al., 2020; Watts et al., 2021). The United States Food and Drug Administration considers ethanol at concentrations of 50–95% mixed with distilled water generally efficient for inactivating several types of viruses (Gerberding et al., 2002). Some studies have shown that ethanol effectively inactivates enveloped viruses, such as influenza virus (Nomura et al., 2021), coronavirus (Harada et al., 2022; Kariwa et al., 2006; Kratzel et al., 2020; Nomura et al., 2021), herpes virus (Tyler & Ayliffe, 1987), and hepatitis B virus (Than et al., 2019). Meanwhile, for sufficient inactivation of non-enveloped viruses, higher ethanol concentrations and longer contact times are required (Belliot et al., 2008; Cromeans et al., 2014; Kramer et al., 2006; Nicole et al., 2018; Ruhlandt et al., 2023; Sattar, 2007; Park et al., 2010). However, the sensitivity of viruses to ethanol has not been quantitatively evaluated.

Machine learning is a branch of artificial intelligence that applies computational algorithms, allowing computers to learn and make decisions based on the data (Keeping Checks on Machine Learning, 2021). It can also be useful for data visualization, showing trends within unstructured data (Zhong et al., 2022). No quantitative analysis has so far been undertaken with machine learning to determine the sensitivity of viruses to ethanol and the most important variables influencing the inactivation of enveloped and non-enveloped viruses exposed to ethanol. The available reviews on the inactivation of viruses using ethanol are primarily based on descriptive analysis (Kampf, 2018; Lin et al., 2020; Sauerbrei, 2020). Consequently, in the present systematic review, we aimed to determine the sensitivity of viruses to ethanol and explore variables that influence virus inactivation when exposed to ethanol by employing machine learning algorithms.

In this study, we first performed a literature review to identify studies on the inactivation of viruses using ethanol. We then analyzed the collected data using machine learning, which included decision trees and random forest algorithms. The decision tree algorithm was developed in the form of tree-like structures to identify explanatory variables (i.e., ethanol concentration and contact time) that could help estimate the output variable. The random forest algorithm was used for classification and regression, based on a model derived from an ensemble of decision trees, to identify the most important variable influencing the output variable of “virus inactivation by ethanol.”

## Methods

**Fig. 1:**
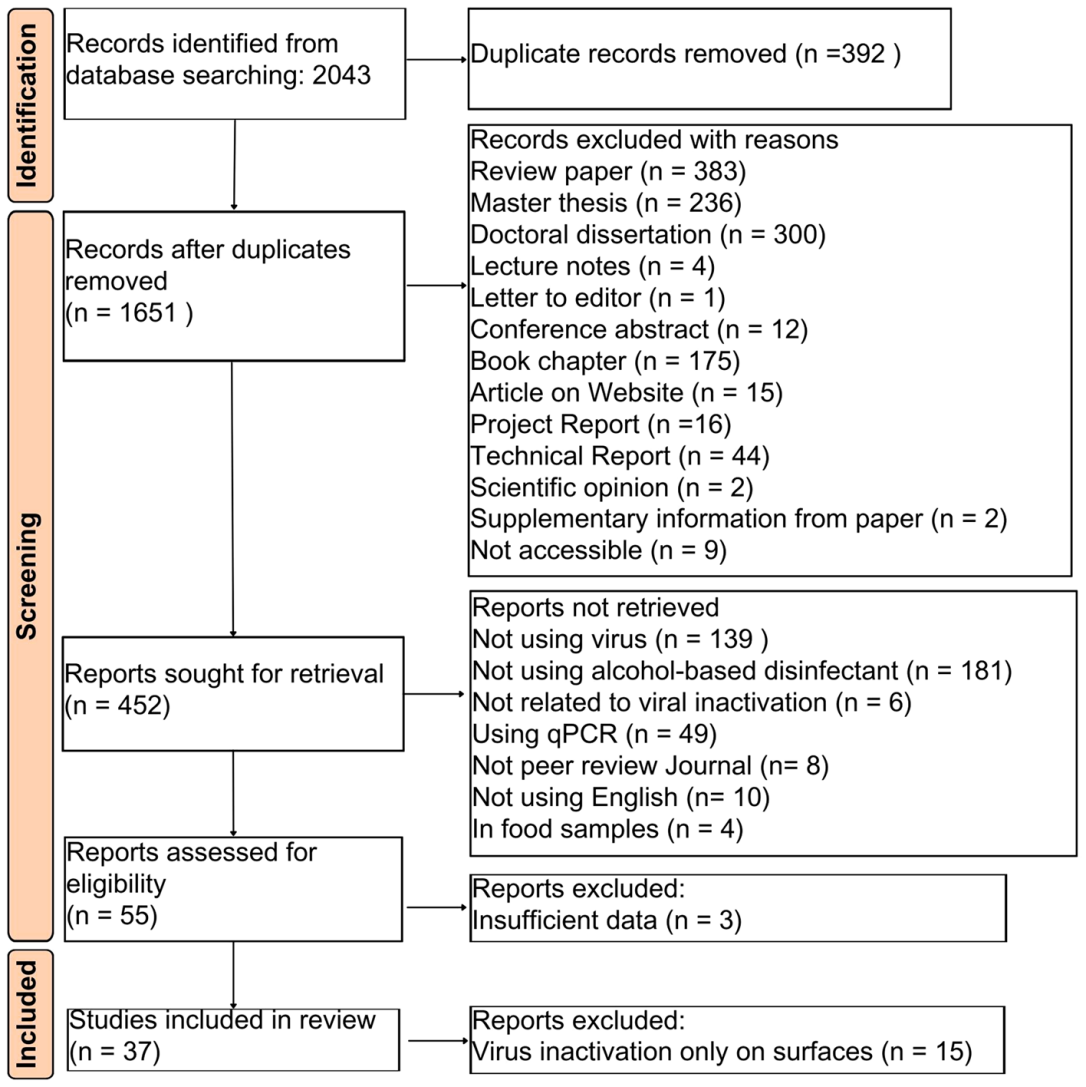
Flowchart depicting the article selection process employed in this systematic review

This systematic review was performed according to Preferred Reporting Items for Systematic Reviews and Meta-Analyses (PRISMA) (Fig. 1 and Supplementary Material 1, Table S1) (Moher et al., 2015; Page et al., 2021).

### Search Strategy

Google Scholar and PubMed databases were used to identify the articles. The search terms were “ethanol” + “inactivation” + “disinfection” + “log reduction” + “log inactivation” + “virus type.” The input keywords for the virus types were “coronavirus,” “norovirus,” “influenza virus,” “hepatitis virus,” “echovirus,” “rotavirus,” “ebolavirus,” and “enterovirus.” Duplicates were removed from the selected articles. Subsequently, the primary screening process was conducted for the selected articles by reading the titles and abstracts. The selected articles were then subjected to full-text screening. Each selected article was then subjected to full-text screening following inclusion criteria, including: (i) were peer-reviewed and in English language; (ii) contained quantitative data for virus inactivation using alcohols in suspensions; and (iii) contained information on inactivation tests in terms of alcohol concentration, contact time, and log_10_ inactivation (log_10_ reduction of viruses were mentioned using cell-culture based experiments like plaque assay and 50% tissue culture infectious dose (TCID50) Assay). The following articles were excluded from the present study: (i) review papers, proceedings, dissertations, thesis, lecture notes, and project reports; (ii) inactivation studies conducted on microorganisms besides viruses or on food samples; (iii) studies that used other active compounds for deactivation; and (iv) studies that performed quantification using qPCR or RT-PCR.

After screening, we extracted data from the texts or tables. We collected data on virus inactivation in terms of the log_10_ reduction value (LRV), which expresses the relative number of viruses eliminated by a disinfectant. We included all LRVs for each virus inactivated at specific alcohol concentrations and contact times. The LRVs were directly extracted from the articles or calculated from log_10_ (*Nt*/*N*0), where *Nt* is the virus concentration at time *t*, and *N*0 is the initial virus concentration. We also used WebPlotDigitizer to extract data if the data were unavailable in the text or tables (Drevon et al., 2017). We recorded the following data from each article: (i) type of virus tested; (ii) ethanol concentration; (iii) ethanol mixture; (iv) exposure time; (v) LRV; (vi) unit; (vii) neutralizer solution; (viii) the test method; (ix) type of host cells; and (x) experimental conditions (Supplementary Material 2). In addition, we determined the risk of bias in the individual studies based on criteria of assessment by the QUIN tool to know the quality of the study (Sheth et al., 2022). Most collected studies (31 articles) had a low risk of bias, while the rest had a medium risk (6 articles). The studies with a medium risk of bias lacked information about the sample size and the statistical analysis (Doultree et al., 1999; Harada et al., 2022; Kurtz et al., 1980; Saknimit et al., 1988; van Bueren et al., 1994; Wolff et al., 2001). The assessment of risk of bias for each study was shown in Supplementary Material 3.

### Data Analysis

Decision trees allow statistical analyses and employ the Classification and Regression Tree (CART) algorithm, which consists of a modeling algorithm represented by a tree-like structure (Therneau & Atkinson, 2022). Decision trees have a root node that shows all datasets, an internal node for making decisions representing the explanatory variables, and a terminal node representing the output variable (Supplementary Material 4, Figure S1). The output variable highlighted the average LRV achieved in the virus inactivation study. We built a decision tree to characterize the datasets of virus inactivation by ethanol based on the *RPART* (Recursive Partitioning and Regression Trees) package in RStudio version 4.2.1 (Therneau & Atkinson, 2022).

An essential step in generating decision trees is selecting the appropriate and most useful splitting criteria for each decision node (Djuris et al., 2013). The Gini impurity is a concept for estimating the optimal split of a decision tree. Gini impurity identifies a particular variable and selects the cutoff point for the variable that minimizes the variance in each of the two subsets resulting from the split (Breiman, 1984). The minimum impurity corresponds to the maximum homogeneity with perfect classification and is associated with a Gini impurity of zero (Breiman, 1984). The Gini impurity (GI) is defined using Eq. (1):

1 $${\text{GI}} = 1 - \sum\nolimits_{i = 1}^{c} {\left( {Pi} \right)^{2} }$$

where *Pi* represents the class percentage of *i* in the node, and index *i* runs from 1 to *C* classes.

In this study, the optimal size of the decision tree was determined based on the complexity parameter (cp) to avoid data overfitting. The cp value with the least cross-validated error was selected and used to prune the trees. The dependent variable for the analysis was LRV, whereas the independent variables were ethanol concentration and contact time.

The principle of random forest is to combine several binary decision trees to make decisions that reduce the possibility of overfitting (Supplementary Material 4, Figure S1) (Zhang et al., 2022). In this study, we used random forest for regression analysis. The regression output is the average estimation for each tree. A random forest was built by applying the bootstrap method, in which we created a random sample with replacement to form the training datasets, called “in-the-bag,” and the test set, called “out-of-bag” (Breiman, 2001). This method allows different possibilities of sample sets to compose each decision tree, and later, the results are aggregated from all the trees to reduce variance. We applied the Random Forest library in RStudio version 4.2.1 with 500 trees (*ntree* = 500). The number of variables selected at each split (*mtry*) in the random forest tree was set to 2.

**Fig. 2:**
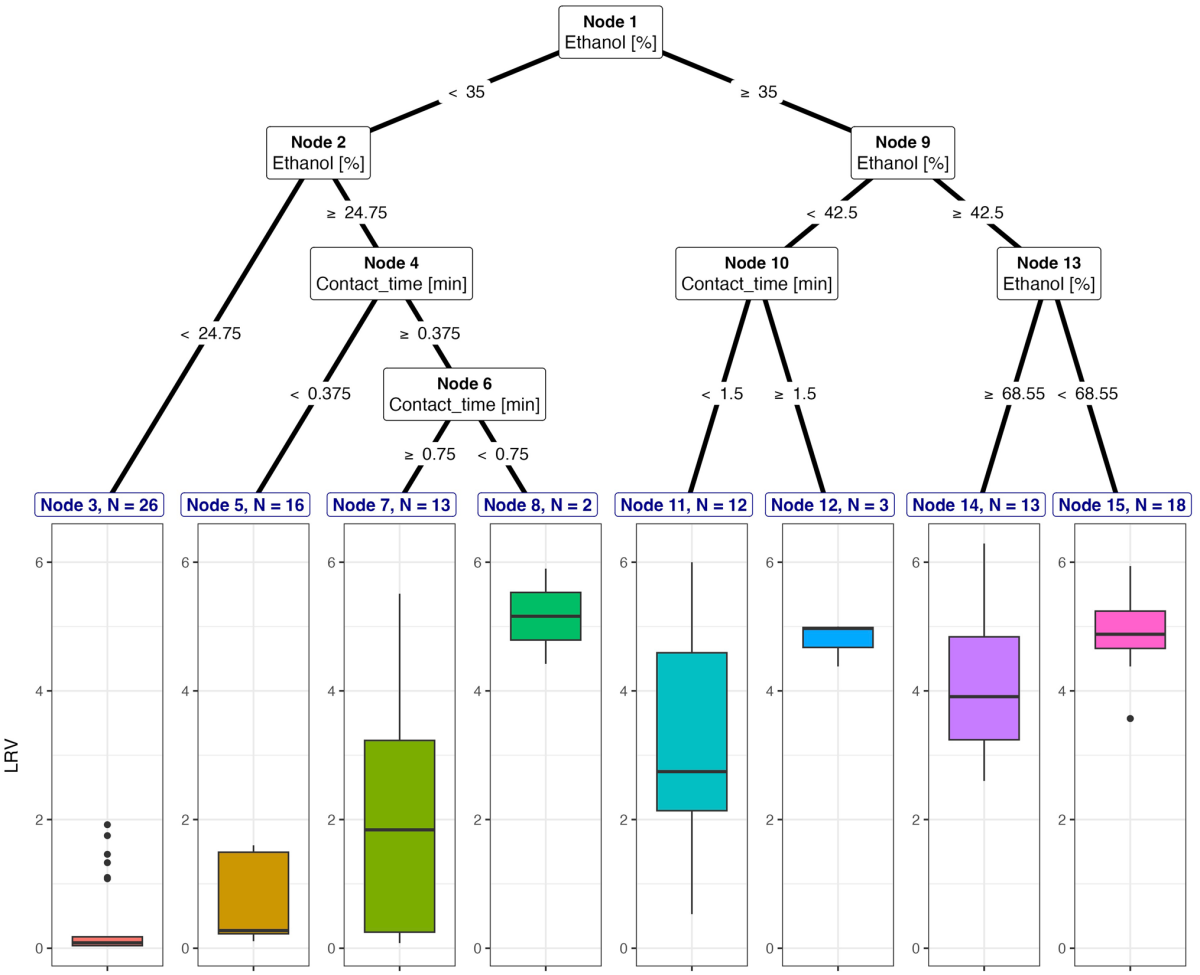
Decision trees for inactivation of enveloped viruses using ethanol in suspension

A random forest was used to estimate the importance of the explanatory variables in the model. The importance of each explanatory was measured using the function “importance” in the “Random Forest” package by permuting each independent variable (Liaw & Wiener, 2002). In the case of random forest, percentage increases in mean square error (%IncMSE) and node purity (%IncNodePurity) were used to rank the variable importance. The %IncMSE value was measured as the increase in the percentage of the mean squared error when the variable was permuted. A larger mean square error for certain variables demonstrates that the input variable has a larger influence on the output variable. The mean squared error (*MSE*) is defined using Eq. (2):

2 $$MSE = \frac{{\sum\nolimits_{i = 1}^{n} {\left( {{{p}} - {{pi}}} \right)^{ \wedge } 2} }}{n}$$

where *n* is the number of out-of-bag samples; *p* is the observed output value; and *pi* is the predicted output obtained using the generated random forest algorithm. The %IncNodePurity value was calculated based on GI by splitting the variables in all the trees. A higher %IncNodePurity value indicates that the variable is more important.

### Statistical Analysis

Virus inactivation data were shown as the mean and standard deviation. Several summary statistics were added, including the median, minimum values, maximum values, the first and third quartiles of the datasets. We used the Mann–Whitney *U* tests to see if there were any differences in the LRV, ethanol concentration, and contact time between enveloped and non-enveloped viruses' datasets. We also performed a statistical analysis based on Spearman correlations (R) to evaluate the correlation between variables (Schober et al., 2018). The values of *p* ≤ 0.05 were considered to have statistical significance, while the values of *p* > 0.05 were statistically nonsignificant. All the analyses were executed using GraphPad version 9.5.1. Results were visualized using RStudio version 4.2.1 and Python version 3.11.

## Results

### Article Selection Process and Data Extraction

We found 2043 relevant articles in the Google Scholar and PubMed databases in the following two searches: first in January 2022 and the second in February 2023. In the end, 37 peer-reviewed articles on inactivation of enveloped and non-enveloped viruses in suspension were included in the final analysis (Fig. 1). Virus inactivation upon exposure to ethanol in suspension was the main focus of our search, considering the presence of sufficient data points in the collected datasets. Finally, datasets on the inactivation of several virus types were categorized primarily into inactivation of enveloped and non-enveloped viruses (Supplementary Material 1, Table S2). We identified the characteristics of the viruses and the standard test methods to assess virus sensitivity to disinfectants in this review based on the available literature (Supplementary Material 1, Tables S3 and S4) (Knipe & Howley, 2021). The total number of data points of enveloped and non-enveloped viruses inactivation by ethanol in suspension was sufficient to be analyzed using a machine learning approach (Cui & Gong, 2018; Leonard et al., 2017). Subsequently, only a dataset of non-enveloped viruses had enough data points to be further categorized based on the presence of organic matter during the inactivation test. Several studies about virus inactivation using ethanol were designed to simulate clean and dirty conditions (Eggers, 1990; Kurtz et al., 1980; Ruhlandt et al., 2023; Su et al., 2021; Uzuner et al., 2018; Wolff et al., 2001; Zonta et al., 2016). The dirty conditions were applied using organic matter such as bovine serum albumin (BSA) and fetal bovine serum (FBS). The use of organic matter in a virucidal efficacy test for a particular disinfectant allowed evaluation of whether organic matter influences the ability of the disinfectant to inactivate viruses (Lin et al., 2020).

### Statistical Summary of the Datasets and the Correlation Between Variables

A preliminary statistical evaluation was performed on the collected datasets for virus inactivation following exposure to ethanol in the suspension test (Supplementary Material 1, Tables S5 and S6). We also visualized the distributions of ethanol concentration, contact time, and LRV as boxplots in the datasets (Supplementary Material 4, Figures S2 and S3). Inactivation of enveloped and non-enveloped viruses by ethanol demonstrated that the ethanol concentrations and contact times were significantly different (Mann–Whitney *U* test, *p* < 0.05), with non-enveloped viruses requiring higher concentrations and longer contact times. The average ethanol concentration of enveloped viruses’ datasets was 38.65% ± 17.19% with the average contact time was 1.39  ± 1.96 min. In contrast, the non-enveloped viruses’ datasets had the mean ethanol concentration and contact time around 72.33% ± 15.74% and 3.72  ± 4.38 min. Furthermore, the achieved LRVs were not significantly different for the inactivation of enveloped and non-enveloped viruses (Mann–Whitney *U* test, *p* = 0.85 and *p* = 0.72, respectively). The mean LRV of enveloped viruses’ datasets was 2.46 ± 2.10, while the average LRV of non-enveloped viruses’ datasets with and without organic matter was 2.42 ± 1.83 and 2.16 ± 1.73, respectively.

We performed the Spearman correlation test to understand the correlation between the variables, particularly the correlation of ethanol and contact time with the achieved LRV. There was a significant positive correlation between ethanol concentrations and the achieved LRV of enveloped viruses, with a Spearman’s correlation coefficient (R) of 0.75 (*p* = 0.001) (Supplementary Material 4, Figure S4). The inactivation data of non-enveloped viruses with and without an organic matter also showed a positive correlation between ethanol concentrations and LRV. However, the correlation coefficients of the non-enveloped virus datasets were lower than that of the enveloped virus inactivation dataset (*R* = 0.24, *p* = 0.002 and *R* = 0.12, *p* = 0.30, respectively). Furthermore, the correlation between contact time and LRVs for the inactivation of enveloped viruses was positive (*R* = 0.38, *p* = 0.01). In all non-enveloped virus datasets, contact time and LRVs were positively correlated (R = 0.21, *p* = 0.006 and *R* = 0.25, *p* = 0.03, respectively) (Supplementary Material 4, Figures S5 and S6). The different correlation results between the enveloped and non-enveloped datasets show that these two groups of viruses have different susceptibilities to ethanol. The applied ethanol concentration correlated more strongly with the log_10_ inactivation, particularly for enveloped viruses.

### Analysis Using Decision Trees and Random Forest Algorithms

Decision trees were developed according to the ethanol concentrations and contact times to estimate the LRV separately for enveloped and non-enveloped viruses in the suspension tests. A lower range of ethanol concentrations and a short contact time were sufficient to inactivate the enveloped viruses. We also considered that adequate virus inactivation happened when the average LRV was around ≥ 4 log_10_ as the minimum required levels of inactivation after disinfection (USEPA, 2020). For the inactivation of enveloped viruses in suspension, the viral load was reduced by more than 2–5 log_10_ by an ethanol concentration of ≥ 35% and an average contact time of ≥ 23 s, and this was observed in approximately 44% of all datasets (*n* = 46) (Fig. 2). Ethanol concentrations of > 35%–65% reduced the viral titer of enveloped viruses by approximately 4 to 5 log_10_. A smaller LRV of less than 3 log_10_ was achieved in the inactivation of enveloped viruses by applying an ethanol concentration range of approximately 35%–42.50% with a contact time of < 1.5 min. The group of enveloped viruses exposed to ethanol with a concentration ranging from > 24.75% to < 35% and an exposure time of approximately ≥ 23 s was observed in 15% (*n* = 15) of all the datasets and reached a mean LRV of 2.56 to 5 log_10_. In contrast, the inactivation of enveloped viruses using lower ethanol concentrations of < 24.75% could only achieve an average LRV of < 1 log_10_ (25% of all datasets, *n* = 26).

**Fig. 3:**
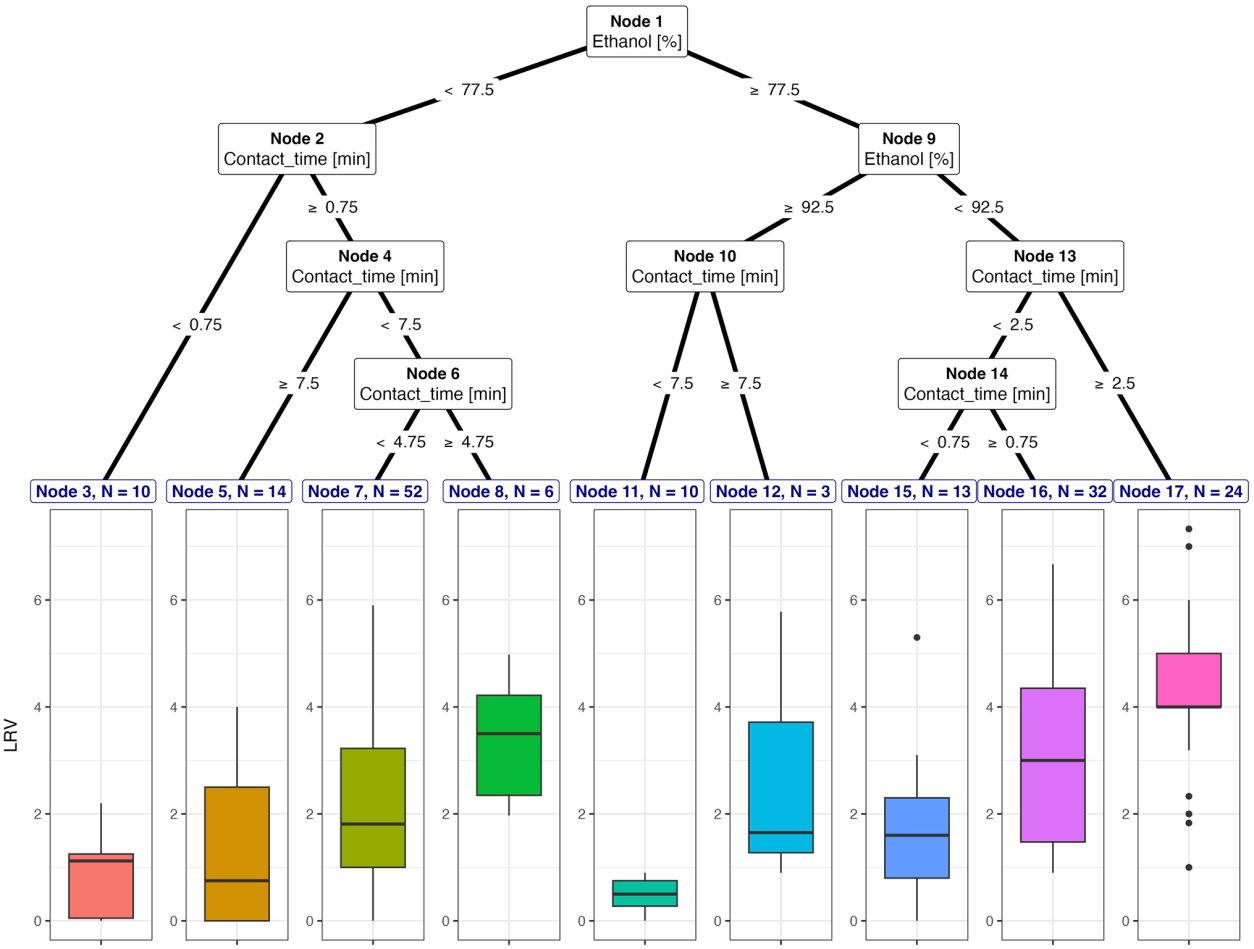
Decision trees for inactivation of non-enveloped viruses using ethanol in suspension with the addition of organic matter

From the results of the inactivation of non-enveloped viruses with and without organic matter, we considered the effective range of ethanol concentrations to be > 65% with contact times of approximately 2 min or longer. Using ethanol concentrations and contact times below this range caused insufficient inactivation of non-enveloped viruses. The inactivation of non-enveloped viruses with the addition of an organic matter was achieved by ≥ 77.50% concentration of ethanol, and this was observed in 50% (*n* = 82) of all datasets with several ranges of ethanol concentration and contact times that reached 2 to 4 log_10_ inactivation (Fig. 3). For the inactivation of non-enveloped viruses, ethanol concentrations with a range of approximately 77.50% to 92.50% and a contact time of about 45 s to ≥ 2.5 min were utilized, and this was predominantly found in 34% of inactivation datasets (*n* = 56), which achieved a reduction of approximately 3.09–4.36 log_10_. However, we also found that a low LRV was reached (approximately less than 1 log_10_) in the inactivation of non-enveloped viruses, and this was found in the inactivation data of HAV using an ethanol concentration of > 92.50% with a contact time range of 1–10 min, and this constituted 0.60% (*n* = 10) of all datasets. Meanwhile, the group of non-enveloped viruses inactivated using < 77.50% ethanol with exposure periods of approximately < 45 s and ≥ 45 s reached average range LRVs of 1.76–3.40 log_10_.

**Fig. 4:**
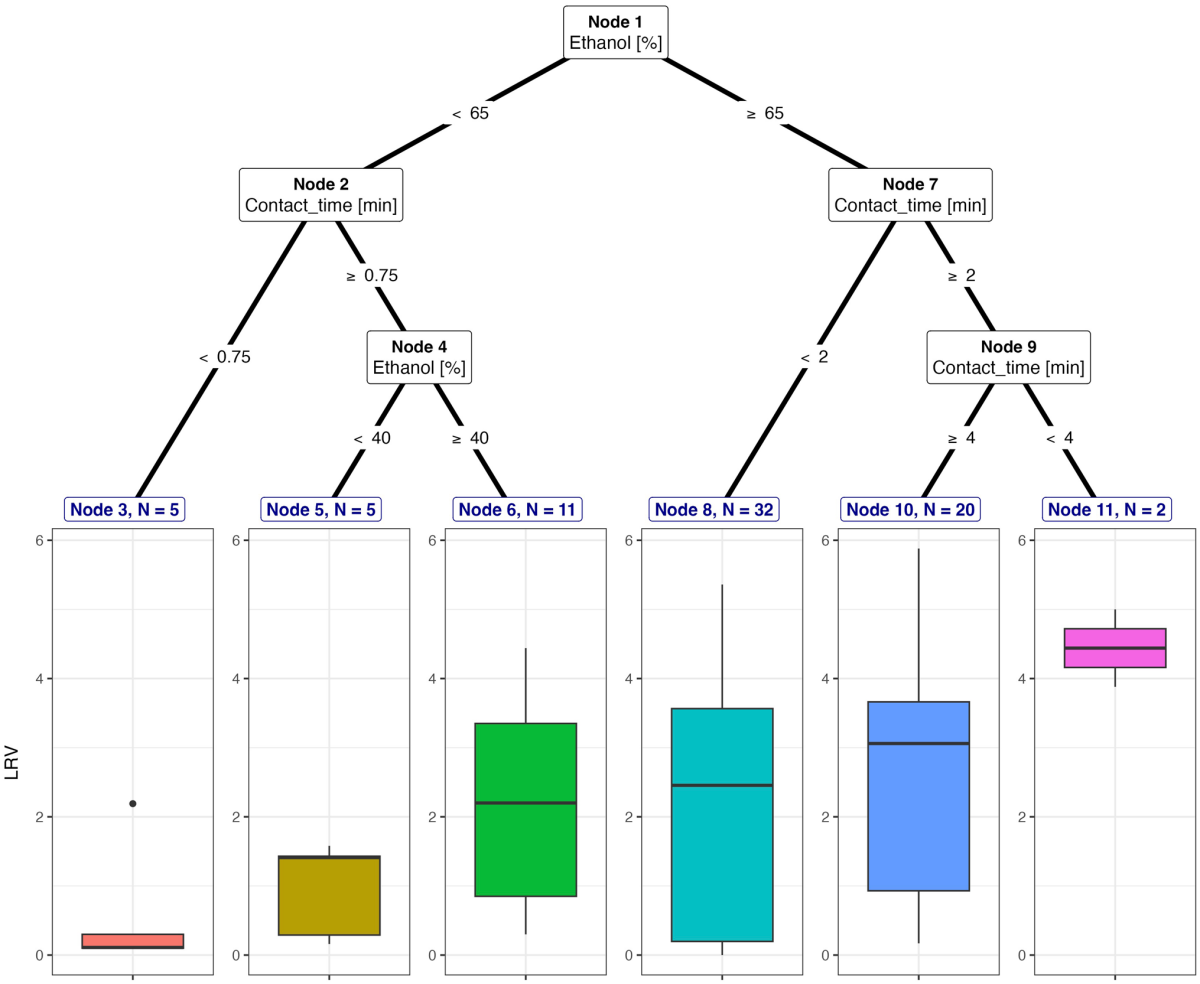
Decision trees for inactivation of non-enveloped viruses using ethanol in suspension without the addition of organic matter

Inactivation of non-enveloped viruses in the suspension test without an organic matter also employed a similar range of ethanol concentration and contact time as required for inactivation with an organic matter (Fig. 4). In this dataset, inactivation using ≥ 65% ethanol with a contact time of > 2 to > 4 min achieved approximately 3 to 4 log_10_ reduction of the viral load of non-enveloped viruses, and this comprised 27% (*n* = 20) of all datasets. We also identified that ≥ 65% ethanol with < 2 min exposure time reduced viral titers by approximately 2.19 log_10_ (43% of all datasets, *n* = 32), except in the case of inactivation of MNV (strain S99) that required a shorter contact time of approximately 0.5 to 1 min with > 65% ethanol to achieve the limit of detection (> 4 log_10_) (3% of all datasets, *n* = 2). In contrast, the ethanol concentration of approximately < 65% and exposure time of < 0.75 min and ≥ 0.75 min could reduce the viral load of non-enveloped viruses by around 0.56 log_10_ and 1.76 log_10_, respectively.

We also applied a random forest algorithm to investigate the variable importance by determining the values of %IncMSE. Overall, ethanol concentration was more important than contact time, based on the %IncMSE value for enveloped viruses (Table 1). Similar results were observed in datasets of inactivation of non-enveloped viruses with the addition of an organic matter, where ethanol concentration was more important than contact time (Table 1). Only datasets of the inactivation of non-enveloped viruses without organic matter addition indicated that the variable of contact time was more important than ethanol concentration. The inactivation datasets for the enveloped viruses suggested that the ethanol concentration and contact time variables had %IncMSE values of 56.58% and 26.20%, respectively. Regarding the inactivation of non-enveloped viruses with organic matter, the ethanol concentration had a %IncMSE value of 31.93%, whereas the contact time had a %IncMSE value of 12.60%. In contrast, the %IncMSE values of ethanol concentration and contact time for inactivation of non-enveloped viruses without an organic matter were 0.62% and 5.93%, respectively.

**Table 1:** %IncMSE and %IncNodePurity values of ethanol concentration and contact time for all datasets

Variable	Enveloped viruses *N*: 103		Non-enveloped viruses (with organic matter) *N*: 164		Non-enveloped viruses (without organic matter) *N*: 75	
	%IncMSE	%IncNodePurity	%IncMSE	%IncNodePurity	%IncMSE	%IncNodePurity
Ethanol concentration	56.58	252.50	31.93	159.37	0.62	36.72
Contact time	26.20	82.10	12.60	102.33	5.93	40.70

*%IncMSE* percentage increase in mean square error, *%IncNodePurity* percentage increase in node purity, *N* total data

In addition to the %IncMSE values, we estimated %IncNodePurity values to evaluate the importance of the variable. Similar trends were observed for the %IncNodePurity values of the ethanol concentration and contact time variables for the inactivation of enveloped viruses (Table 1). The %IncNodePurity values for ethanol concentration and contact time were 252.50% and 82.10%, respectively. A higher value of %IncNodePurity was observed for ethanol concentration than that for contact time in the datasets of inactivation of non-enveloped viruses with organic matter addition; values of %IncNodePurity were approximately 159.37% and 102.33%, respectively. For the dataset of inactivation of non-enveloped viruses without organic matter, the %IncNodePurity values for ethanol concentration and contact time were 36.72% and 40.70%, respectively. The %IncMSE and %IncNodePurity values indicated that ethanol concentration is a more important variable than contact time for virus inactivation, especially for the inactivation of enveloped and non-enveloped viruses with the addition of an organic matter. The different amounts of data led to the different results of %IncMSE and %IncNodePurity in the datasets of non-enveloped viruses with and without organic matter. A larger observation in the dataset of non-enveloped viruses with the addition of organic matter allows for more robust modeling and may lead to more reliable estimates of the variables. Meanwhile, smaller datasets had more variation, which can result in lower %IncMSE and %IncNodePurity. As a result, the dataset of non-enveloped virus inactivation with the addition of organic matter gave more reliable results related to the most important variables for virus inactivation under ethanol exposures. These results imply that the selected ethanol concentration influenced the speed of virus inactivation more than the contact time. However, the virus type must also be considered. Some viruses require adequate contact time to effectively reduce their viral loads, specifically several types of non-enveloped viruses. Inactivation studies on the hepatitis A virus (HAV) using 95% ethanol achieved a reduction of about 2 log_10_ only after a contact time of around 10 min (Wolff et al., 2001). In less than 10 min, the average LRV reached less than 1 log_10_ reduction (Wolff et al., 2001). Poliovirus and adenovirus required more than 5 min of contact time to reach 4 log_10_ of viral reduction using 80% ethanol (Eggers, 1990).

## Discussion

This study aimed to quantitatively analyze the sensitivity of viruses to ethanol and estimate the importance of explanatory variables that influence virus inactivation following exposure to ethanol. This was done by employing decision trees and random forest algorithms on virus inactivation datasets from previous studies. Different ethanol sensitivities of the viruses were observed, depending on the selected ethanol concentration and contact time. Sufficient inactivation of enveloped viruses was achieved with lower ethanol concentrations and shorter contact times. In contrast, inactivation of non-enveloped viruses required higher ethanol concentrations and longer exposure times. The results also revealed that the ethanol concentration was a more important variable than the contact time for enveloped and non-enveloped virus inactivation with organic matter. A different finding was observed in the datasets of inactivation of non-enveloped viruses without an organic matter, where contact time was a more important variable than ethanol concentration. The dataset of non-enveloped viruses inactivation without an organic matter used a lower average ethanol concentration that can lead to the lower inactivation of viruses. As a result, adequate contact time is essential during the inactivation of those non-enveloped viruses.

The main reason for the differences in the required range of ethanol concentration is related to virus structure, which can lead to different responses to disinfectants. Enveloped viruses contain lipid membranes on their outer surface derived from host cells and thus can be easily destroyed by ethanol, whereas non-enveloped viruses lack lipid membranes and are less susceptible to ethanol (van Engelenburg et al., 2002; Yoshikawa et al., 2012). Ethanol can form hydrogen bonds with the lipid bilayer, decreasing the ordering of the lipid hydrocarbon chains that allow ethanol to penetrate the lipid bilayer easily (Patra et al., 2006). Recent studies on the effect of ethanol on coronaviruses that belong to enveloped viruses have revealed that ethanol accelerates lipid membrane disintegration, which can initiate the destruction of the viral spike protein and trigger the release of internal materials (Basak & Deb, 2021; Das et al., 2021). In non-enveloped viruses, ethanol may alter the structure or intermolecular interactions in virus proteins and induce coagulation of capsid proteins, which causes loss of cellular function and requires longer contact time than in enveloped viruses (Ali et al., 2021; Yoshikawa et al., 2012; Yoshizawa et al., 2014).

The sensitivity of non-enveloped viruses to chemicals has high variability, which may be the reason for the differing results of sensitivity to ethanol among non-enveloped viruses (Zhou, 2022). An ethanol concentration of 40% with a contact time of 1 min can effectively inactivate vaccinia virus (VACV), duck hepatitis B virus (DHBV), and vesicular stomatitis virus (VSV) (Rabenau et al., 2010; Sauerbrei et al., 2012; Zimmer et al., 2013). Meanwhile, several non-enveloped viruses like MNV and HAV need contact times around 5 min using 70% ethanol to reach more than 4 log_10_ reductions (Cromeans et al., 2014; Song et al., 2022; Park et al., 2010). Other non-enveloped viruses, such as human enterovirus and poliovirus, require higher ethanol concentrations and contact times longer than 5 min (Chang et al., 2013; Eggers, 1990). A study that compared the inactivation of two MNV strains reported that they had different susceptibilities to ethanol (Min et al., 2022). The varied sizes of the non-enveloped samples may have also affected the level of sensitivity to ethanol. Non-enveloped viruses with larger particle diameters were considered more sensitive to chemical disinfectants than smaller ones (Sattar, 2007). However, other studies revealed that MNV, a small non-enveloped virus, achieved a 4 log_10_ reduction when exposed to 70% ethanol with a contact time of approximately 30 s to 1 min in the suspension test (Cromeans et al., 2014; Imai et al., 2020; Park et al., 2010). Conversely, adenovirus serotypes 8, 19, and 37, which are large non-enveloped viruses, did not achieve sufficient inactivation when subjected to similar ethanol concentrations and contact times (Uzuner et al., 2018).

The sensitivity of the virus to ethanol also differs depending on the application conditions, such as the disinfectant ratio, availability of interfering substances, temperature, and pH. Most published virus inactivation studies have mentioned interfering substances such as organic matter. The effectiveness of ethanol can be reduced during disinfection with available organic matter because ethanol may first interact with the hydrophobic binding sites on the organic matter (Devi et al., 2009; Ulrich, 2013). However, several studies have not completely described the application conditions, especially the temperature, pH, and relative humidity. Most studies noted that the inactivation experiment was performed at approximately 22 ± 2°C or used the term “room temperature,” with no accurate information about conditions being provided. A study reported that the virucidal activity of ethanol improved under the increasing temperature between 20 and 25°C and the rising pH around 9, suggesting that temperature and pH are essential for the inactivation of viruses during disinfection (Ruhlandt et al., 2023). Humidity is also an important factor influencing disinfectant penetration into virus suspensions (Lin et al., 2020). We suggest that each study's data related to the experimental conditions should be more specific. More comprehensive virus inactivation datasets should be obtained and analyzed, leading to a more in-depth investigation of factors influencing virus inactivation in various areas.

This study has several limitations. First, data related to virus inactivation by ethanol are limited because ethanol is mainly used for disinfection with short exposure times, and it is particularly used for inactivation of enveloped viruses. Therefore, testing a large range of ethanol concentrations and various contact times has not been the primary focus in research. Second, many inactivation studies have also utilized laboratory strains of viruses, but whether the results can represent human viruses remains to be clarified. Third, standard test methods to assess virus sensitivity to disinfectants sometimes differ slightly between studies depending on the virus type. The use of specific virucidal testing standards, including the European Norms (EN) standard, the American Society for Testing Materials (ASTM) standard, or other guidelines from a specific institution, such as the German Association for the Control of Viral Diseases (DVV) or the Robert Koch Institute (RKI) result in varied results (Ionidis et al., 2016; Sattar et al., 2002; Steinmann & Wolff, 2007). These methods have some differences in the testing requirements, such as the type of virus, volume ratio of viruses to disinfectants, type of neutralizer, and type of interfering substance. Fourth, in the present review, we focused only on the ethanol solution containing no other additives. However, in commercial hand sanitizers, ethanol is commonly added to substances such as moisturizers, emollients, and fragrances, which can also influence the strength of ethanol in inactivating viruses (Abuga & Nyamweya, 2021).

In conclusion, this systematic review is the first to use machine learning algorithms to identify sensitivity of viruses to ethanol and to explore the most important variable influencing inactivation of viruses by ethanol. This review presents an evaluation of the range of ethanol concentrations and contact times required for inactivating enveloped and non-enveloped viruses. The use of a random forest algorithm also confirmed the importance of the ethanol concentration in inactivation of viruses, which can be used as a guideline to ensure the effectiveness of the disinfection process. Previous studies have reported that repeated exposure to disinfectants, such as chlorine and lime, can lead to the development of less sensitive virus populations (Kadoya et al., 2022; Oishi et al., 2022; Rachmadi et al., 2018). Therefore, continuously reviewing disinfectant efficacy against viruses, such as that of ethanol, is essential, considering the potency of viruses to evolve and become more resistant to disinfectants.

### Supplementary Information

Below is the link to the electronic supplementary material.

Supplementary file1 (PDF 217 KB)

Supplementary file2 (XLSX 41 KB)

Supplementary file3 (PDF 147 KB)

Supplementary file4 (PDF 943 KB)
