# Supplementary material for: Sensitivity Evaluation of Enveloped and Non-enveloped Viruses to Ethanol Using Machine Learning: A Systematic Review
Source: Food Environ Virol. 2023 Dec 5;16(1):1–13. doi: 10.1007/s12560-023-09571-2 (PMC10963467; doi:10.1007/s12560-023-09571-2)
Supplement: Supplementary file 1 — Supplementary file1 (PDF 217 KB) [file 12560_2023_9571_MOESM1_ESM.pdf]

SUPPLEMENTARY TABLES

Sensitivity evaluation of enveloped and non-enveloped viruses to ethanol using machine learning: a systematic review

Aken Puti Wanguyun[1], Wakana Oishi[2], Daisuke Sano[1,2*]

[1]Department of Frontier Science for Advanced Environment, Graduate School of Environmental Studies, Tohoku University, Sendai, Japan
[2]Department of Civil and Environmental Engineering, Graduate School of Engineering, Tohoku University, Sendai, Japan

***Correspondence:** daisuke.sano.e1@tohoku.ac.jp

Table S1. PRISMA 2020 checklist [1]

Section and topic	Item	Checklist item	Location where item is reported
**TITLE**			
Title	1	Identify the report as a systematic review	Page 1
**ABSTRACT**			
Structured summary	2	Provide a structured summary including, as applicable: background; objectives; data sources; methods; results; conclusions and implications of key findings; conclusions	Page 1
**INTRODUCTION**			
Rationale	3	Describe the rationale for the review in the context of what is already known.	(Introduction) 4th paragraph
Objectives	4	Provide an explicit statement of the questions and objectives being addressed with reference to their key elements.	(Introduction) 4th paragraph
**METHODS**			
Eligibility criteria	5	Specify study characteristics and report characteristics used as criteria for eligibility	(Methods) 1st to 2nd paragraph
Information sources	6	Describe all information sources in the search and date last searched.	(Methods) 1st to 2nd paragraph
Search strategy	7	Present full electronic search strategy for at least one database, including any limits used, such that it could be repeated.	(Methods) 1st to 2nd paragraph
Study selection	8	State the process for selecting studies (i.e., screening, eligibility, included in systematic review)	(Methods) 1st to 2nd paragraph
Data collection process	9	Describe method of data extraction from reports and any processes for obtaining and confirming data from investigators.	(Methods) 1st to 2nd paragraph
Data items	10	List and define all variables for which data were sought and any assumptions and simplifications made.	(Methods) 1st to 2nd paragraph
Study risk of bias assessment	11	Describe methods used for assessing risk of bias of individual studies.	(Methods) 1st to 2nd paragraph
**RESULTS**			

Study selection	12	Describe the results of the search and selection process, from the number of records identified in the search to the number of studies included in the review, ideally using a flow diagram.	Fig. 1
Study characteristics	13	For each study, present characteristics for which data were extracted.	(Results) 1st paragraph
Risk of bias in studies	14	Present data on risk of bias of each study	Supplementary Material 3
**DISCUSSION**			
Summary of evidence	15	Summarize the main results (including an overview of concepts, themes, and types of evidence available), link to the review questions and objectives.	(Discussion) 1st to 4th paragraph
Limitations	16	Discuss the limitations of the review.	(Discussion) 5th paragraph
Conclusions	17	Provide a general interpretation of the results with respect to the review questions and objectives, and potential implications for future study.	(Discussion) 6th paragraph
**FUNDING**			
Funding	18	Describe sources of funding for the study.	Available in funding section

Table S2. List of enveloped and non-enveloped viruses in the datasets

Type of virus	Virus name	Virus abbreviation	Ref
Enveloped viruses	Vaccinia virus strain Lister Elstree	VACV	[2]
	Modified vaccinia virus ankara	MVA	[2]
	Severe acute respiratory syndrome coronavirus 2	SARS-CoV-2	[3–8]
	Influenza A virus	IAV	[4, 9]
	Vesicular stomatitis virus	VSV	[10]
	Rabies virus	RABV	[10]
	Hepatitis B virus	HBV	[11]
	Mouse hepatitis virus	MHV	[12]
	Duck hepatitis B virus	DHBV	[13]
	Human immunodeficiency virus	HIV	[14]
	Canine coronavirus	CCV	[12]
Non-enveloped viruses	Feline calicivirus	FCV	[15–22]
	Murine norovirus	MNV	[17–19, 23, 24]
	Rotavirus	RoV	[25]
	Bovine rotavirus	BRV	[26]
	Enterovirus	EV	[27, 28]
	Hepatitis E virus	HEV	[29]
	Hepatitis A virus	HAV	[30, 31]
	Reovirus	Reo	[25]
	Adenovirus	AdV	[25, 32, 33]
	Human papillomavirus	HPV	[34]
	Astrovirus	AstV	[26]
	Echovirus	Echo	[25]
	Simian vacuolating virus	SV	[34]
	Canine calicivirus	CaCV	[20]
	Aichi virus	AiV	[18]
	Poliovirus	PV	[25, 28, 35]
	Porcine enteric calicivirus	PEC	[18]
	Tulane virus	TuV	[18, 36]
	Canine parvovirus	CPV	[12]
	Kilham rat virus	KRV	[12]

Table S3. Characteristics of viruses identified in studies on inactivation in suspension

Family	Viruses included in this review	Nucleocapsid morphology	Virion morphology	Size (nm)	Genome	Host
**Enveloped viruses**						
Rhabdoviridae (-ssRNA)	VSV	Coiled helical filaments	Bullet shaped	70–170	Linear, 11–15kb	Vertebrate, insect, plant
Orthomyxoviridae (-ssRNA)	IAV	Helical filaments	Pleomorphic, spherical	80–200	Linier, 10–15 kb	Vertebrate
Coronaviridae (+ssRNA)	SARS-CoV-2, MHV	Helical	Spherical	80–160	Linear, 28–31 kb	Vertebrate
Filoviridae (-ssRNA)		Helical filaments	Bacilliform, filamentous	80–100	Linear, 19 kb	Vertebrate
Poxviridae (dsDNA)	VACV, MVA	Ovoid	Ovoid	220–450	Linear, 130–375 kb	Vertebrate, insect
Retroviridae (+ssRNA)	HIV	Spherical, rod, or cone shaped	Spherical	80–100	Linear RNA dimer, 7–13 kb	Vertebrate
Hepadnaviridae (RNA and DNA reverse transcribing viruses)	HBV	Icosahedral	Spherical	42–50	Double-stranded circular DNA, 3–4 kb	Vertebrate
**Non-enveloped viruses**						
**Large non-enveloped viruses (50–100 nm)**						
Papillomaviridae (dsDNA)	HPV	Icosahedral	Icosahedral	52–55	ds circular, 7–8 kb	Vertebrate
Polyomaviridae (dsDNA)	SV	Icosahedral	Icosahedral	40–45	ds circular, 5 kb	Vertebrate
Adenoviridae (dsDNA)	AdV	Icosahedral	Icosahedral	70–100	Linear, 26–45 kb	Vertebrate
Reoviridae (dsRNA)	RoV	Icosahedral	Icosahedral	60-80	Linear, 18.5 kb	Vertebrate
**Small non-enveloped viruses (less than 50 nm)**						
Caliciviridae (+ssRNA)	MNV, FCV, TuV, PEC	Icosahedral	Icosahedral	35–40	Linear, 7-9 kb	Vertebrate
Astroviridae (+ssRNA)	AstV	Isometric	Isometric	28–30	Linear, 6–7 kb	Vertebrate

Picornaviridae (+ssRNA)	EV, HAV, AiV, Echo, PV	Icosahedral	Icosahedral	20–30	Linear, 7–8 kb	Vertebrate
Parvovirus (± ssDNA)	CPV, KRV	Icosahedral	Icosahedral	18-26	Linear, 5 kb	Vertebrate
Hepeviridae (+ssRNA)	EV	Icosahedral	Icosahedral	27-34	Linear, 7.2 kb	Vertebrate

Note: single-stranded RNA; dsRNA, double-stranded RNA; ssDNA, single-stranded DNA; dsDNA, double-stranded

Table S4. Methods for evaluating virucidal activity of ethanol in suspension

Methods	Virus	Ratio virus and ethanol	Neutralizer	Interfering substances	Ref
Quantitative suspension test	MNV	1:9	DMEM	-	[24]
Quantitative suspension test	AiV, FCV, MNV, TuV, PEC	1:9	EMEM plus 10% FBS	-	[18]
Quantitative suspension test	FCV	1:9	HBSS	-	[15]
Quantitative suspension test	PV, EV	1:4	Sodium hyposulfite, Polysorbate 80, lecithin, glycine, and Dey-Engley buffer	30 g/L BSA	[28]
Quantitative suspension test	MNV, FCV	1:9	10% FBS in PBS	-	[17]
Quantitative suspension test	PV, Echo, AdV, Reo, RoV	1:8	Ice-cold PBS	2% BSA	[25]
Quantitative suspension test	TuV	1:6, 1:7, 1:9	Opti-MEM with 2% FBS	-	[36]
Quantitative suspension test	HAV	1:8	Ice-cold culture medium	2% BSA or FCS	[31]
Quantitative suspension test	SARS-CoV-2	1:8	DMEM	0.3% BSA	[3]
Quantitative suspension test	SARS-CoV-2	1:8	DMEM	3% BSA	[5]
Quantitative suspension test	VSV	1:8	MEM with 5% FBS	FBS	[10]
Quantitative suspension test	HBV	1:4	DMEM	-	[11]

Quantitative suspension test	RABV		G-MEM containing 10% FCS and antibiotic antimycotic	-	[37]
Quantitative suspension test	IAV	3:7	DMEM	-	[38]
Quantitative suspension test	HIV	1:9	RPMI 1640 medium supplemented with 10% FCS	-	[14]
Quantitative suspension test	BRV, AstV	1:9	Skimmed milk	calf serum	[26]
Quantitative suspension test	CPV, KRV, MHV-2, MHV-N	-	MEM containing 2% FCS	-	[12]
Quantitative suspension test	FCV, CaCV	-	DMEM	-	[20]
Quantitative suspension test	FCV	-	5% sucrose in TES buffer	-	[21]
Quantitative suspension test (The Institutional Review Board of Kyoto Prefectural University of Medicine (approval no. ERB-C-1593)	IAV	1:19	SCLDP medium	-	[9]
DVV/RKI guideline	DHBV	1:8	Ice-cold cell culture medium	FCS	[13]
ASTM standard E-1052	EV	1:9	DMEM supplemented with 2% FCS	-	[27]

ASTM standard E-1052-11	HAV	1:9	Serum-free DMEM	-	[30]
ASTM standard E-1052-11	SARS-CoV-2, IAV	1:9	DMEM	-	[4]
ASTM standard E 1052-96	FCV, MNV	1:9	FBS	-	[19]
Quantitative Robert Koch-Institut, Berlin, Germany and DVV (Suspension test)	FCV	1:8	EMEM	-	[22]
Robert Koch-Institut, Berlin, Germany and DVV (Suspension test)	HPV, SV40	1:8	Ice-cold cell culture medium	0.3 g/L BSA	[34]
EN 14476	AdV-8, AdV-19, AdV-37	1:8	Cold DMEM containing 2% FBS	0.3 g/L BSA	[32]
EN 14476	MNV, FCV	1:8	Ice-cold cell culture medium	0.3% BSA	[16]
EN 14476	AdV-5	1:8	EMEM	0.3 g/l BSA	[33]

Note: No data (-); BSA, bovine serum albumin; DMEM, Dulbecco's modified Eagle medium; EMEM, Eagle’s Minimum Essential Medium with Hanks’salts; FBS, fetal bovine serum; FCS, fetal calf serum, G-MEM, Glasgow’s minim essential medium; HBSS, Hanks Balanced Salt Solution; MEM, modified Eagle medium; PBS, phosphate buffered saline; RPMI, Roswell Park Memorial Institute; SCLDP, soybean–casein digest broth prepared with lecithin and polysorbate 80.

Table S5. Statistical analysis of the dataset related to inactivation of enveloped viruses by ethanol in suspension

Virus group	Virus type	Total data	Variable	Mean	Standard deviation	Median	Min	Max	Q1	Q3
Enveloped viruses	VACV, MVA, SARS-CoV-2, IAV, VSV, RABV, HBV, MHV, DHBV, HIV, CCV	103	Ethanol concentration (%)	38.65	17.19	34	10	70	24.75	50
			Contact time (min)	1.39	1.96	1	0.25	10	0.25	1
			LRV	2.46	2.10	1.92	0.03	6.29	0.21	4.54

Note: LRV, log10 reduction value; Q1, the first quartile; Q3, the third quartile

Table S6. Statistical analysis of the dataset related to inactivation of non-enveloped viruses by ethanol in suspension

Virus group	Addition of organic matter	Virus type	Total data	Variable	Mean	Standard deviation	Median	Min	Max	Q1	Q3
Non-enveloped viruses	Yes	EV, PV, AdV, Echo, HPV, SV, AstV, HEV	164	Ethanol concentration (%)	73.79	14.52	77.50	20	97	70	80
				Contact time (min)	4.14	4.91	2	0.50	15	1	5
				LRV	2.42	1.83	2	0	7.33	1	4
	No	FCV, MNV, TuV, AiV, PEC, HAV, CaCV, Reo	75	Ethanol concentration (%)	69.13	17.81	70	30	90	55	90
				Contact time (min)	2.78	2.69	1	0.50	10	1	5
				LRV	2.16	1.73	2.19	0	5.88	0.30	3.59

Note: LRV, log10 reduction value; Q1, the first quartile; Q3, the third quartile

References:

1. Page MJ, McKenzie JE, Bossuyt PM, et al (2021) The PRISMA 2020 Statement: An Updated Guideline for Reporting Systematic Reviews. The BMJ 372: https://doi.org/10.1136/bmj.n71
2. Rabenau HF, Rapp I, Steinmann J (2010) Can Vaccinia Virus be Replaced by MVA Virus for Testing Virucidal Activity of Chemical Disinfectants? BMC Infect Dis 10:185. https://doi.org/https://doi.org/10.1186/1471-2334-10-185
3. Kratzel A, Todt D, V'kovski P, et al (2020) Inactivation of SARS-CoV-2 by Hand Rub Formulations. Emerging Infectious Diseases 26:1592–1595. https://doi.org/10.1101/2020.03.10.986711v1
4. Nomura T, Nazmul T, Yoshimoto R, et al (2021) Ethanol Susceptibility of SARS-CoV-2 and Other Enveloped Viruses. Biocontrol Sci 26:177–180. https://doi.org/https://doi.org/10.4265/bio.26.177
5. Xiling G, Yin C, Ling W, et al (2021) In vitro Inactivation of SARS-CoV-2 by Commonly used Disinfection Products and Methods. Sci Rep 11. https://doi.org/10.1038/s41598-021-82148-w
6. Hirose R, Bandou R, Ikegaya H, et al (2021) Disinfectant Effectiveness against SARS-CoV-2 and Influenza Viruses Present on Human Skin: Model-based Evaluation. Clinical Microbiology and Infection 27:1042.e1-1042.e4. https://doi.org/10.1016/j.cmi.2021.04.009
7. Huang Y, Xiao S, Song D, Yuan Z (2022) Evaluating The Virucidal Activity of Four Disinfectants against SARS-CoV-2. Am J Infect Control 50:319–324. https://doi.org/10.1016/j.ajic.2021.10.035
8. Chan KH, Sridhar S, Zhang RR, et al (2020) Factors Affecting Stability and Infectivity of SARS-CoV-2. Journal of Hospital Infection 106:226–231. https://doi.org/10.1016/j.jhin.2020.07.009
9. Bandou R, Hirose R, Nakaya T, et al (2022) Higher Viral Stability and Ethanol Resistance of Avian Influenza A(H5N1) Virus on Human Skin. Emerg Infect Dis 28:639–649. https://doi.org/10.3201/eid2803.211752
10. Zimmer B, Summermatter K, Zimmer G (2013) Stability and Inactivation of Vesicular Stomatitis Virus, A Prototype Rhabdovirus. Vet Microbiol 162:78–84. https://doi.org/10.1016/j.vetmic.2012.08.023
11. Than TT, Jo E, Todt D, et al (2019) High Environmental Stability of Hepatitis B Virus and Inactivation Requirements for Chemical Biocides. Journal of Infectious Diseases 219:1044–1048. https://doi.org/10.1093/infdis/jiy620
12. Saknimit M, Inatsuki I, Sugiyama Y, Yagami K-I (1988) Virucidal Efficacy of Physico-chemical Treatments Against Coronaviruses and Parvoviruses of Laboratory Animals. Exp Anim 37:341–345. https://doi.org/https://doi.org/10.1538/expanim1978.37.3_341
13. Sauerbrei A, Schacke M, Glück B, et al (2012) Does Limited Virucidal Activity of Biocides include Duck Hepatitis B Virucidal Action? BMC Infect Dis 12. https://doi.org/10.1186/1471-2334-12-276
14. Van Bueren J, Larkin DP, Simpson RA (1994) Inactivation of Human Immunodeficiency Virus Type 1 by Alcohols. Journal of Hospital Infection 28:137–148. https://doi.org/https://doi.org/10.1016/0195-6701(94)90140-6

15. Nicole W, Douglas D, Xueling S, Stephen H (2018) Persistent Virucidal Activity in an Alcohol-Based Sanitizer Formulation (ProtecTeaV) for Potential Use against Norovirus. Microbiology & Infectious Diseases 2. https://doi.org/10.33425/2639-9458.1029
16. Zonta W, Mauroy A, Farnir F, Thiry E (2016) Comparative Virucidal Efficacy of Seven Disinfectants Against Murine Norovirus and Feline Calicivirus, Surrogates of Human Norovirus. Food Environ Virol 8:1–12. https://doi.org/10.1007/s12560-015-9216-2
17. Park GW, Barclay L, Macinga D, et al (2010) Comparative Efficacy of Seven Hand Sanitizers against Murine Norovirus, Feline Calicivirus, and GII.4 Norovirus. J Food Prot 73:2232–2238. https://doi.org/https://doi.org/10.4315/0362-028X-73.12.2232
18. Cromeans T, Park GW, Costantini V, et al (2014) Comprehensive Comparison of Cultivable Norovirus Surrogates in Response to Different Inactivation and Disinfection Treatments. Appl Environ Microbiol 80:5743–5751. https://doi.org/10.1128/AEM.01532-14
19. Tung G, Macinga D, Arbogast J, Jaykus LA (2013) Efficacy of Commonly used Disinfectants for Inactivation of Human Noroviruses and Their Surrogates. J Food Prot 76:1210–1217. https://doi.org/10.4315/0362-028X.JFP-12-532
20. Duizer E, Bijkerk P, Rockx B, et al (2004) Inactivation of Caliciviruses. Appl Environ Microbiol 70:4538–4543. https://doi.org/10.1128/AEM.70.8.4538-4543.2004
21. Doultree JC, Druce JD, Birch CJ, et al (1999) Inactivation of Feline Calicivirus, A Norwalk Virus Surrogate. journal of Hospital Infection 41:51–57. https://doi.org/https://doi.org/10.1016/S0195-6701(99)90037-3
22. Gehrke C, Steinmann J, Goroncy-Bermes P (2004) Inactivation of Feline Calicivirus, A Surrogate of Norovirus (Formerly Norwalk-like Viruses), by Different Types of Alcohol in Vitro and in Vivo. Journal of Hospital Infection 56:49–55. https://doi.org/10.1016/j.jhin.2003.08.019
23. Imai K, Hagi A, Inoue Y, et al (2020) Virucidal Efficacy of Olanexidine Gluconate as a Hand Antiseptic Against Human Norovirus. Food Environ Virol 12:180–190. https://doi.org/10.1007/s12560-020-09422-4
24. Belliot G, Lavaux A, Souihel D, et al (2008) Use of Murine Norovirus as A Surrogate to Evaluate Resistance of Human Norovirus to Disinfectants. Appl Environ Microbiol 74:3315–3318. https://doi.org/10.1128/AEM.02148-07
25. Eggers HJ (1990) Experiments on Antiviral Activity of Hand Disinfectants. Some Theoretical and Practical Considerations. Zentralblatt fur Bakteriologie 273:36–51. https://doi.org/10.1016/S0934-8840(11)80238-0
26. Kurtz JB, Lee TW, Parsons AJ (1980) The Action of Alcohols on Rotavirus, Astrovirus and Enterovirus. Journal of Hospital Infection 1:321–325. https://doi.org/https://doi.org/10.1016/0195-6701(80)90008-0
27. Chang SC, Li WC, Huang KY, et al (2013) Efficacy of Alcohols and Alcohol-based Hand Disinfectants Against Human Enterovirus 71. Journal of Hospital Infection 83:288–293. https://doi.org/10.1016/j.jhin.2012.12.010
28. Su Y, Han J, Li J, et al (2021) Resistance of Poliovirus 1 and Enterovirus A71 against Alcohol and Other Disinfectants. J Virol Methods 298. https://doi.org/10.1016/j.jviromet.2021.114292
29. Behrendt P, Friesland M, Wißmann JE, et al (2022) Hepatitis E Virus is Highly Resistant to Alcohol-based Disinfectants. J Hepatol 76:1062–1069. https://doi.org/10.1016/j.jhep.2022.01.006

30. Song M, Hossain MI, Jung S, et al (2022) Comparison of Virucidal Efficacy of Sodium Hypochlorite, Chlorine Dioxide, Peracetic Acid, and Ethanol Against Hepatitis A Virus by Carrier and Suspension Tests. Int J Food Microbiol 363. https://doi.org/10.1016/j.ijfoodmicro.2021.109506
31. Wolff MH, Schmitt J, Rahaus M, Kgnig A (2001) Hepatitis A Virus: A Test Method for Virucidal Activity. journal of Hospital Infection 48:18–22. https://doi.org/https://doi.org/10.1016/S0195-6701(01)90007-6
32. Uzuner H, Karadenizli A, Er DK, Osmani A (2018) Investigation of The Efficacy of Alcohol-based Solutions on Adenovirus Serotypes 8, 19 and 37, Common Causes of Epidemic Keratoconjunctivitis, After an Adenovirus Outbreak in Hospital. Journal of Hospital Infection 100:30–36. https://doi.org/10.1016/j.jhin.2018.05.011
33. Ruhlandt M, Becker B, Paulmann D, et al (2023) Impact of Concentration, Temperature and pH on The Virucidal Activity of Alcohols against Human Adenovirus. Am J Infect Control. https://doi.org/10.1016/j.ajic.2023.01.014
34. Hufbauer M, Wieland U, Gebel J, et al (2021) Inactivation of Polyomavirus SV40 as Surrogate for Human Papillomaviruses by Chemical Disinfectants. Viruses 13. https://doi.org/10.3390/v13112207
35. Kramer A, Galabov AS, Sattar SA, et al (2006) Virucidal Activity of a New Hand Disinfectant with Reduced Ethanol Content: Comparison with Other Alcohol-based Formulations. Journal of Hospital Infection 62:98–106. https://doi.org/10.1016/j.jhin.2005.06.020
36. Arthur SE, Gibson KE (2015) Physicochemical Stability Profile of Tulane Virus: A Human Norovirus Surrogate. J Appl Microbiol 119:868–875. https://doi.org/10.1111/jam.12878
37. Wu G, Selden D, Fooks AR, Banyard A (2017) Inactivation of Rabies Virus. J Virol Methods 243:109–112. https://doi.org/10.1016/j.jviromet.2017.02.002
38. Jeong EK, Bae JE, Kim IS (2010) Inactivation of Influenza A Virus H1N1 by Disinfection Process. Am J Infect Control 38:354–360. https://doi.org/10.1016/j.ajic.2010.03.003
