# Supplementary material for: Sensitivity Evaluation of Enveloped and Non-enveloped Viruses to Ethanol Using Machine Learning: A Systematic Review
Source: Food Environ Virol. 2023 Dec 5;16(1):1–13. doi: 10.1007/s12560-023-09571-2 (PMC10963467; doi:10.1007/s12560-023-09571-2)
Supplement: Supplementary file 3 — Supplementary file3 (PDF 147 KB) [file 12560_2023_9571_MOESM3_ESM.pdf]

SUPPLEMENTARY MATERIAL 3

Sensitivity evaluation of enveloped and non-enveloped viruses to ethanol using machine learning: a systematic review

Aken Puti Wanguyun[1], Wakana Oishi[2], Daisuke Sano[1,2*]

[1]Department of Frontier Science for Advanced Environment, Graduate School of Environmental Studies, Tohoku University, Sendai, Japan
[2]Department of Civil and Environmental Engineering, Graduate School of Engineering, Tohoku University, Sendai, Japan

***Correspondence:** daisuke.sano.e1@tohoku.ac.jp

A. The assessment of risk of bias

The assessment of risk of bias based on QUIN tool consisted of 12 criteria (Sheth et al., 2022) . The final score for each study was calculated to classify the risk of bias (>70% = low, 50–70% = medium, and, <50%=high) using the following formula:

$$\text{Final score} = \frac{(Total\ score\ x\ 100)}{(2\ x\ the\ number\ of\ criteria\ applicable)}$$

B. Criteria of assessment by QUIN tool

No	Criteria	No	Criteria
1	Clearly stated aims/objectives	7	Randomization
2	Detailed explanation of sample size calculation	8	Method of measurement of outcome
3	Detailed explanation of sampling technique	9	Outcome assessor details
4	Details of comparison group	10	Blinding
5	Detailed explanation of methodology	11	Statistical analysis
6	Operator details	12	Presentation of results

C. The score of quality assessment using QUIN tool

No	Study	Criteria												Final score
		1	2	3	4	5	6	7	8	9	10	11	12	
1	Ruhlandt *et al*. (2023)	2	2	2	2	2	NA	NA	NA	NA	NA	1	2	92.86
2	Bandou *et al*. (2022)	2	2	2	2	2	NA	NA	NA	NA	NA	1	2	92.86
3	Harada *et al*. 2022	2	0	2	2	2	NA	NA	NA	NA	NA	0	1	64.29
4	Huang *et al*. (2022)	2	0	2	2	2	NA	NA	NA	NA	NA	0	2	71.43
5	Song *et al*. (2022)	2	2	2	2	2	NA	NA	NA	NA	NA	2	2	100.00
6	Behrendt *et al*. (2022)	2	2	2	2	1	NA	NA	NA	NA	NA	0	2	78.57
7	Hufbauer *et al*. (2021)	2	2	2	2	2	NA	NA	NA	NA	NA	1	2	92.86
8	Nomura *et al*. (2021)	2	0	2	2	2	NA	NA	NA	NA	NA	1	2	78.57
9	Su *et al*. (2021)	2	2	2	2	1	NA	NA	NA	NA	NA	0	2	78.57

10	Xiling *et al*. (2021)	2	2	2	2	2	NA	NA	NA	NA	NA	1	2	92.86
11	Kratzel *et al*. (2020)	2	2	2	2	2	NA	NA	NA	NA	NA	2	2	100.00
12	Chan *et al*. (2020)	2	2	2	2	1	NA	NA	NA	NA	NA	1	2	85.71
13	Imai *et al*. (2020)	2	2	2	2	2	NA	NA	NA	NA	NA	2	2	100.00
14	Than *et al*. (2019)	2	2	2	2	2	NA	NA	NA	NA	NA	2	2	100.00
15	Nicole *et al*. (2018)	2	2	2	2	1	NA	NA	NA	NA	NA	1	2	85.71
16	Uzuner *et al*. (2018)	2	0	2	2	2	NA	NA	NA	NA	NA	2	2	85.71
17	Wu *et al*. (2017)	2	2	2	2	1	NA	NA	NA	NA	NA	1	1	78.57
18	Arthur and Gibson, (2015)	2	2	2	2	1	NA	NA	NA	NA	NA	2	2	92.86
19	Zonta *et al*. (2015)	2	2	2	2	2	NA	NA	NA	NA	NA	2	1	92.86
20	Cromeans *et al*. (2014)	2	2	2	2	2	NA	NA	NA	NA	NA	2	2	100.00
21	Chang *et al*. (2013)	2	2	2	2	2	NA	NA	NA	NA	NA	1	2	92.86
22	Tung *et al*. (2013)	2	2	2	2	2	NA	NA	NA	NA	NA	2	2	100.00
23	Sauerbrei *et al*. (2012)	2	2	2	2	2	NA	NA	NA	NA	NA	1	2	92.86
24	Zimmer *et al*. (2012)	2	2	2	2	2	NA	NA	NA	NA	NA	1	2	92.86
25	Jeong *et al*. (2010)	2	2	2	2	2	NA	NA	NA	NA	NA	0	2	85.71
26	Park *et al*. (2010)	2	2	2	2	2	NA	NA	NA	NA	NA	2	2	100.00

27	Rabenau *et al*. (2010)	2	2	2	2	2	NA	NA	NA	NA	NA	1	2	92.86
28	Duizer *et al*. (2004)	2	2	2	2	2	NA	NA	NA	NA	NA	1	2	92.86
29	Gehrke *et al*. (2004)	2	2	2	2	2	NA	NA	NA	NA	NA	1	2	92.86
30	Belliot *et al*. (2008)	2	0	2	2	1	NA	NA	NA	NA	NA	1	2	71.43
31	Kramer *et al*. (2006)	2	2	2	2	2	NA	NA	NA	NA	NA	1	2	92.86
32	Wolff *et al*. (2001)	2	0	2	2	1	NA	NA	NA	NA	NA	0	2	64.29
33	Doultree *et al*. (1999)	2	0	2	2	1	NA	NA	NA	NA	NA	0	2	64.29
34	Bueren *et al*. (1994)	2	0	2	2	1	NA	NA	NA	NA	NA	0	2	64.29
35	Eggers, (1990)	2	0	2	2	2	NA	NA	NA	NA	NA	0	2	71.43
36	Saknimit *et al*. (1988)	2	0	2	2	1	NA	NA	NA	NA	NA	0	2	64.29
37	Kurtz *et al*. (1980)	2	0	2	2	1	NA	NA	NA	NA	NA	0	2	64.29

Note: Number of criteria is following the criteria in B. Criteria of assessment by QUIN tool; NA, not applicable.

Reference:

Sheth, V. H., Shah, N. P., Jain, R., Bhanushali, N. & Bhatnagar, V. (2022). Development and Validation of A Risk-of-bias Tool for Assessing in Vitro Studies Conducted in Dentistry: The QUIN. *The Journal of Prosthetic Dentistry*. https://doi.org/https://doi.org/10.1016/j.prosdent.2022.05.019
