# Supplementary material for: Sensitivity Evaluation of Enveloped and Non-enveloped Viruses to Ethanol Using Machine Learning: A Systematic Review
Source: Food Environ Virol. 2023 Dec 5;16(1):1–13. doi: 10.1007/s12560-023-09571-2 (PMC10963467; doi:10.1007/s12560-023-09571-2)
Supplement: Supplementary file 4 — Supplementary file4 (PDF 943 KB) [file 12560_2023_9571_MOESM4_ESM.pdf]

SUPPLEMENTARY FIGURES

Sensitivity evaluation of enveloped and non-enveloped viruses to ethanol using machine learning: a systematic review

Aken Puti Wanguyun[1], Wakana Oishi[2], Daisuke Sano[1,2]*

[1]Department of Frontier Science for Advanced Environment, Graduate School of Environmental Studies, Tohoku University, Sendai, Japan
[2]Department of Civil and Environmental Engineering, Graduate School of Engineering, Tohoku University, Sendai, Japan

***Correspondence:** daisuke.sano.e1@tohoku.ac.jp

a.

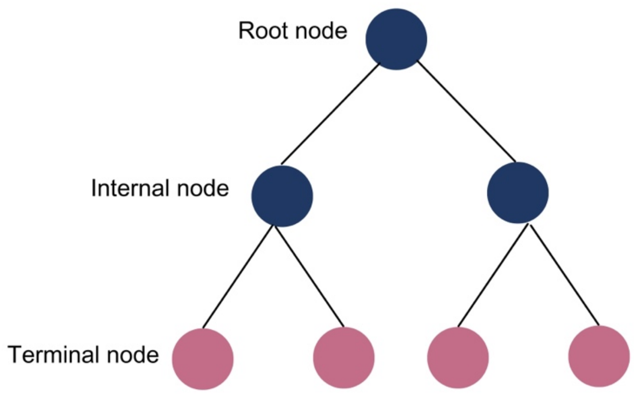

b.

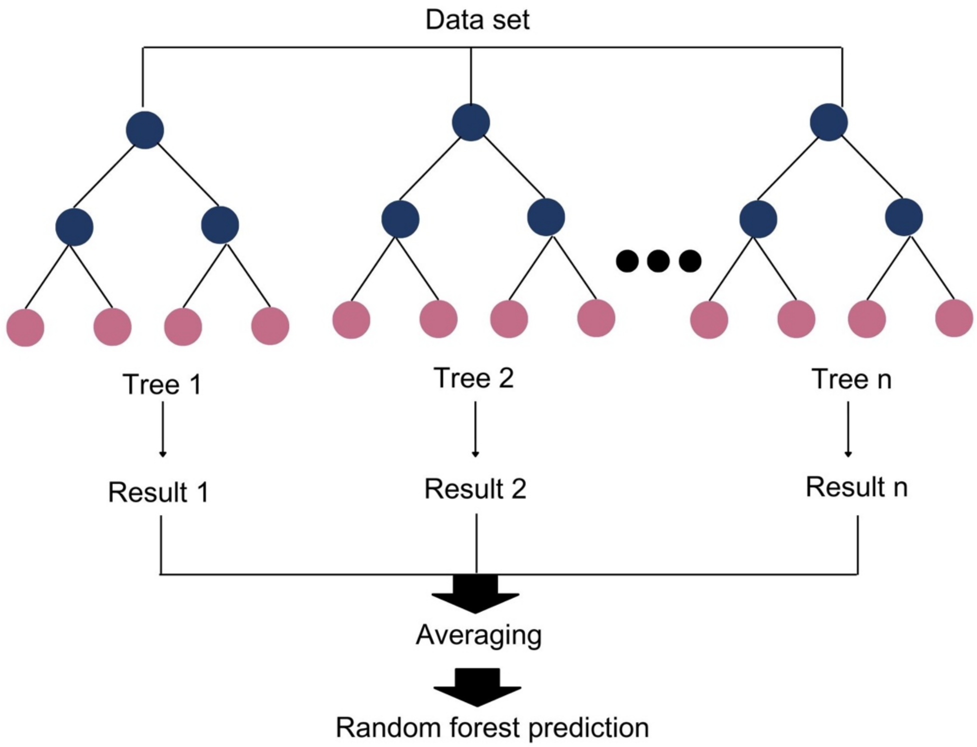

Figure S1. a. Decision trees diagram, b. Random forest diagram

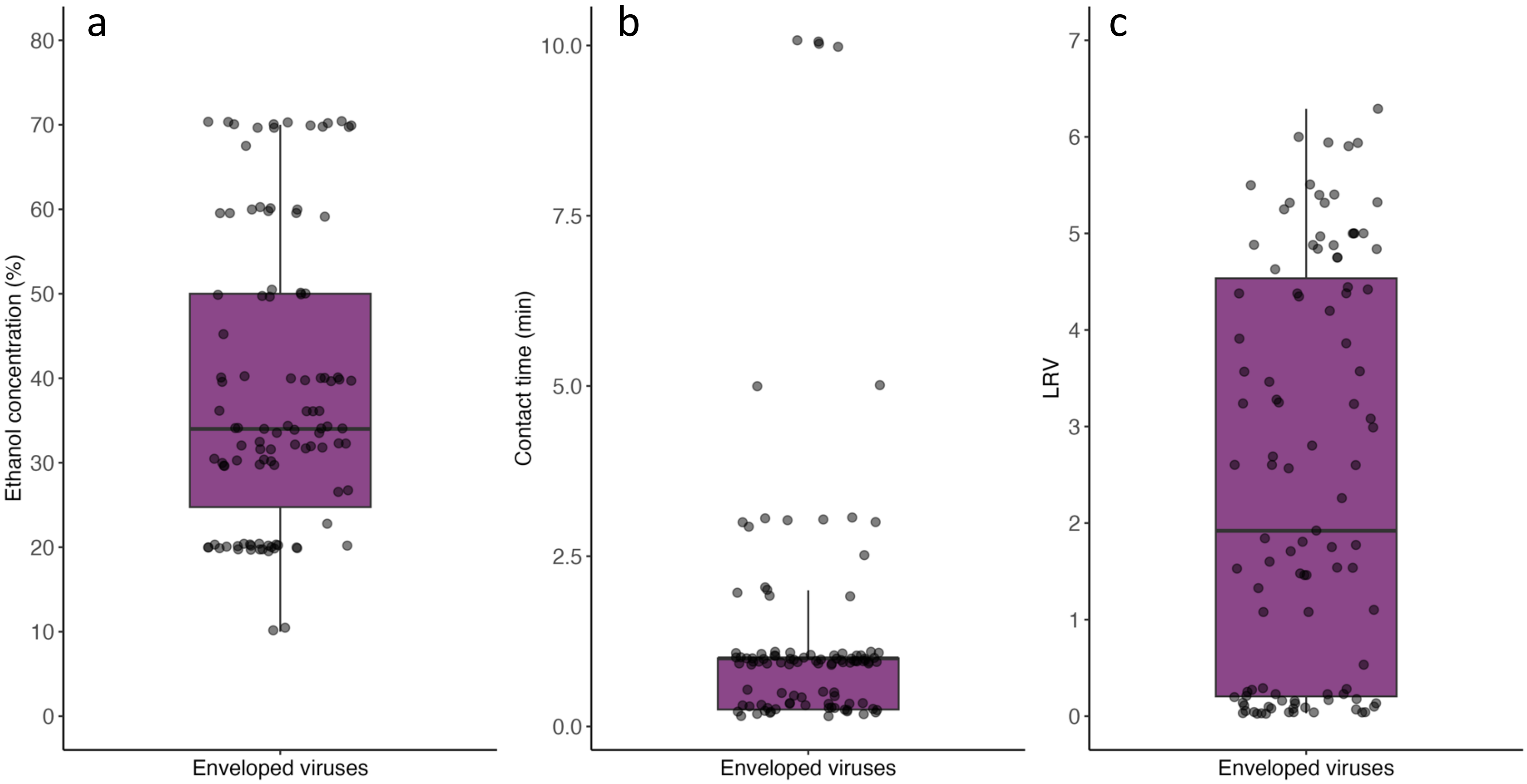

Figure S2. Some variables ([a] ethanol concentration, [b] contact time, and [c] LRV related to inactivation of enveloped viruses by ethanol in suspension
Note: LRV, log10 reduction value

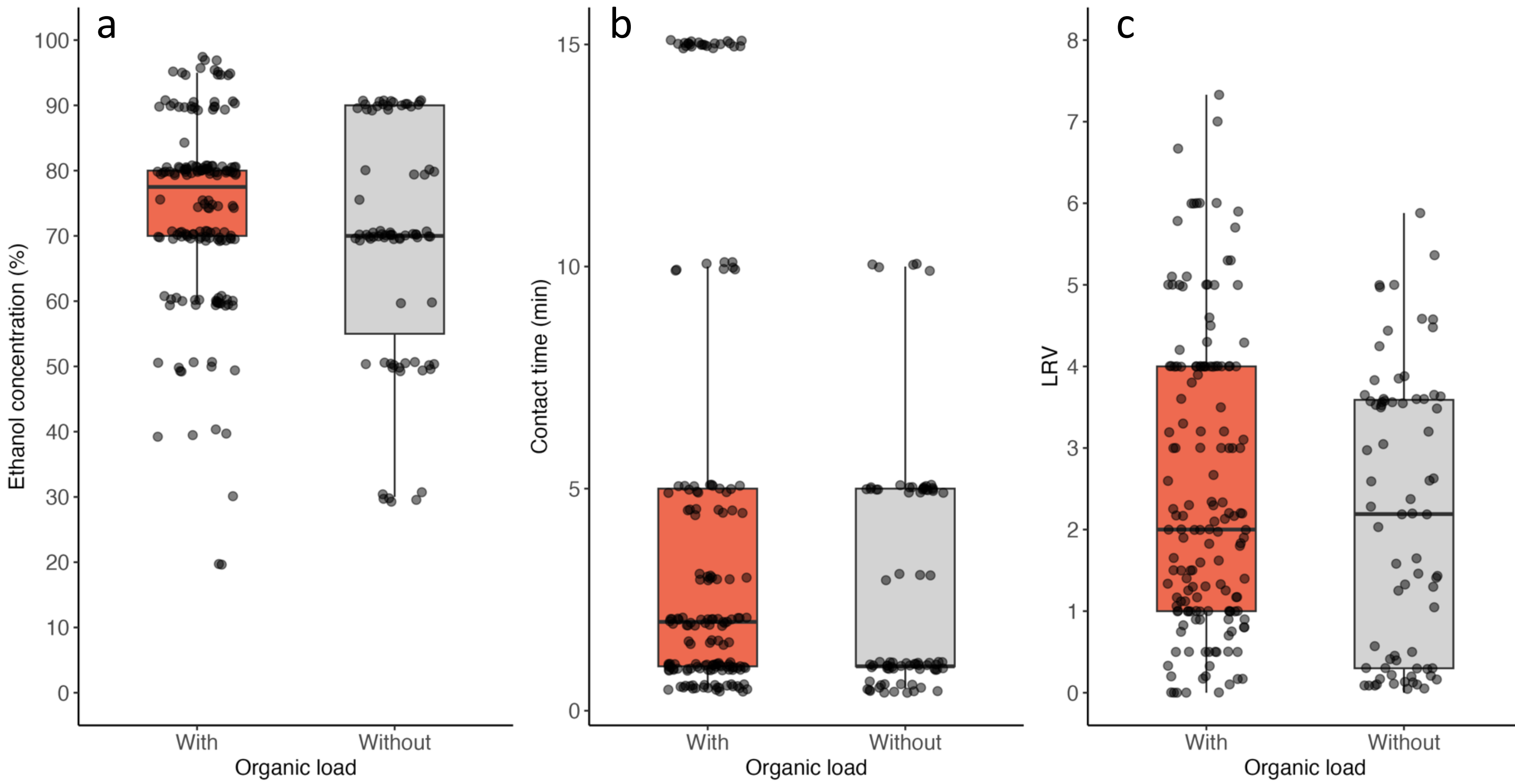

Figure S3. Some variables ([a] ethanol concentration, [b] contact time, and [c] LRV related to inactivation of non-enveloped viruses by ethanol in suspension

Note: LRV, log10 reduction value

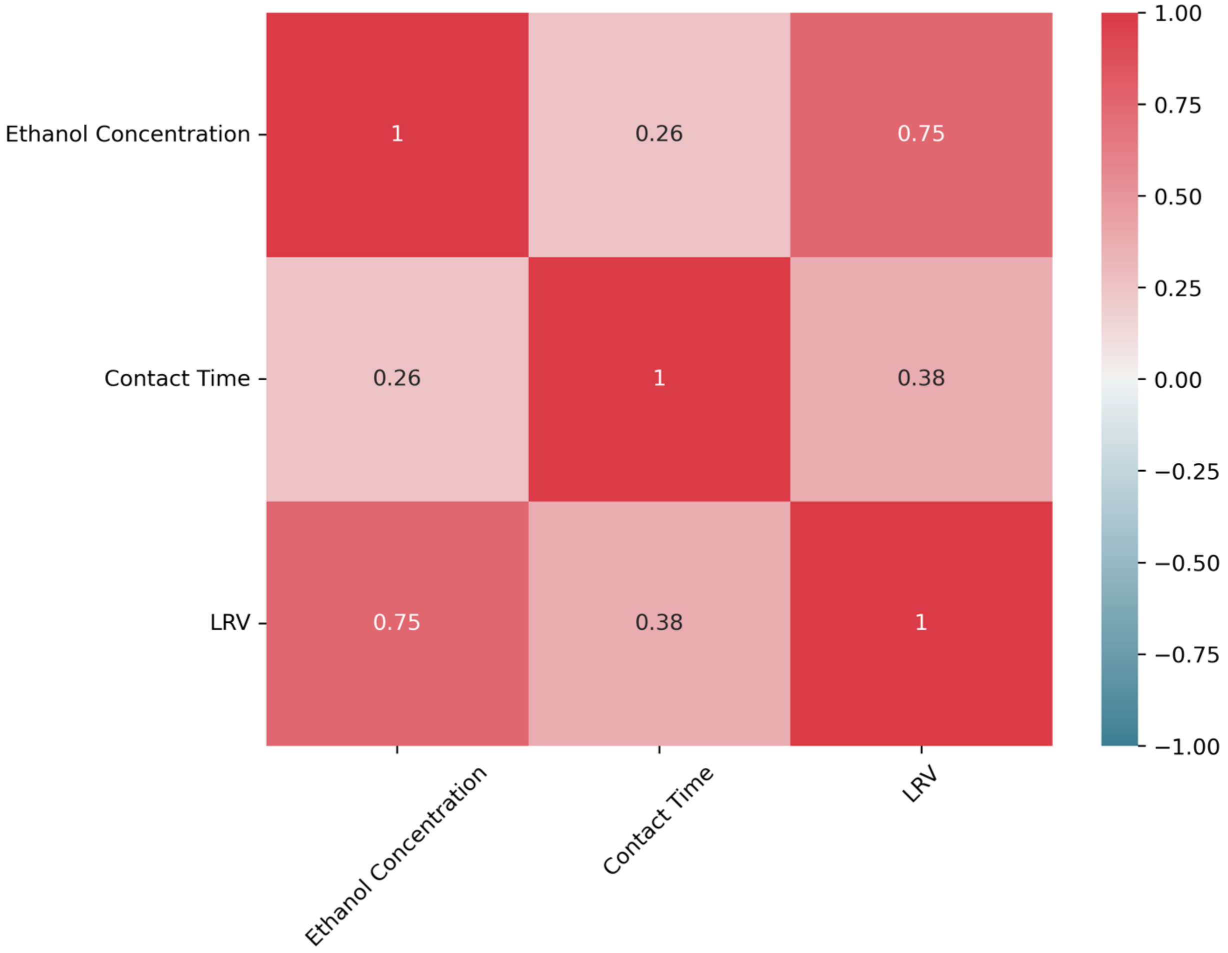

Figure S4. Correlation between multiple variables of inactivation of enveloped viruses by ethanol

Note: LRV, log10 reduction value

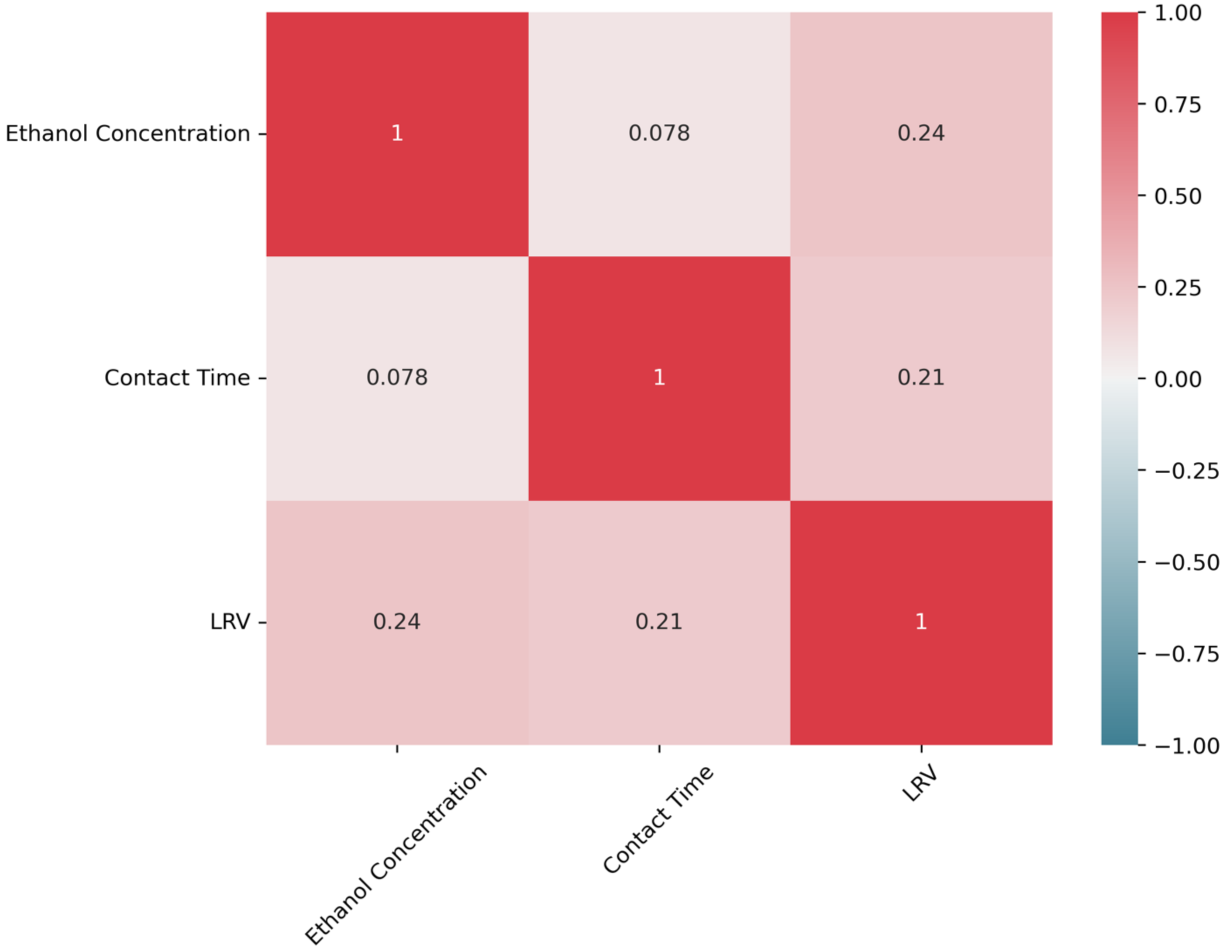

Figure S5. Correlation between multiple variables of inactivation of non-enveloped viruses by ethanol with addition of organic matter

Note: LRV, log10 reduction value

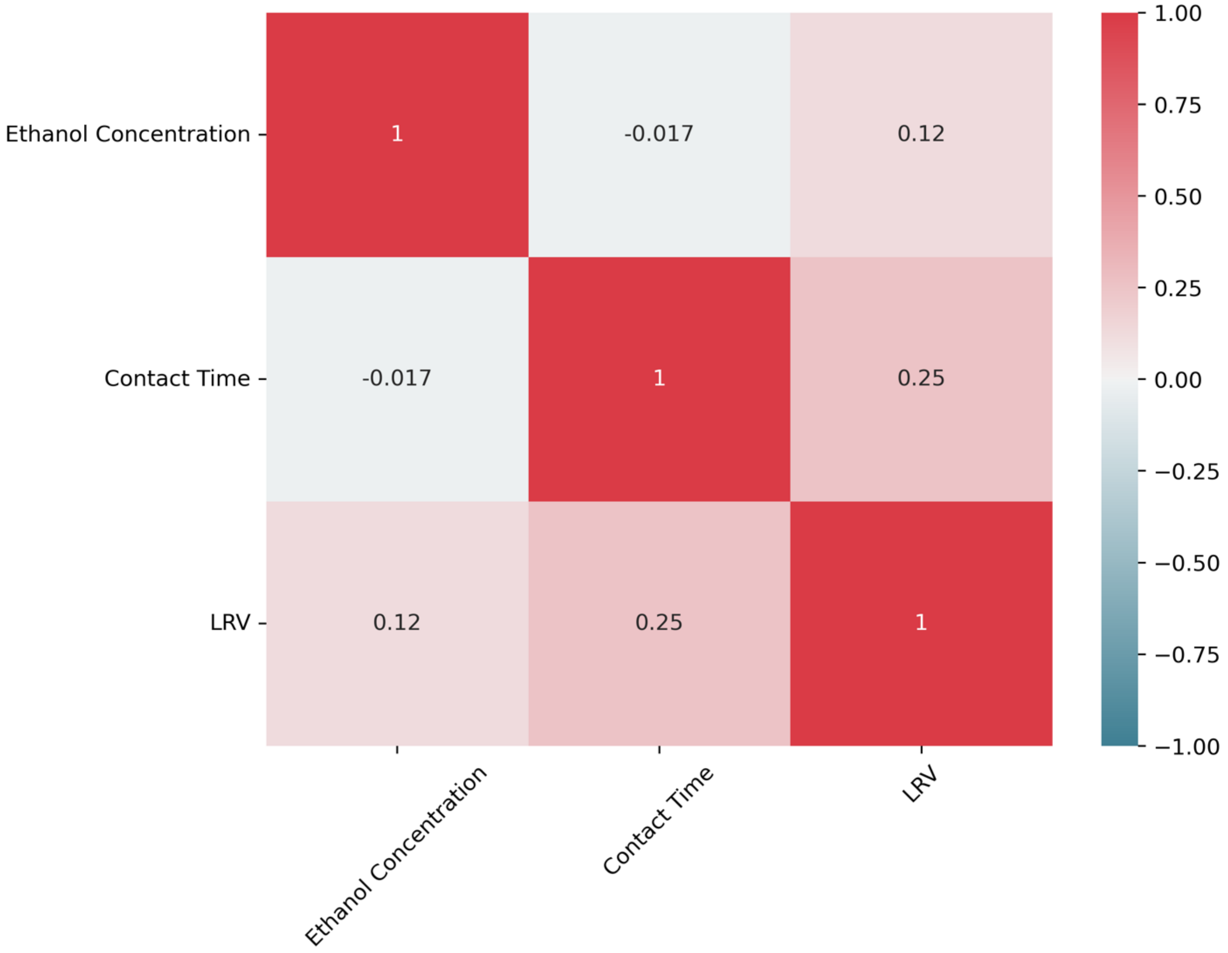

Figure S6. Correlation between multiple variables of inactivation of non-enveloped viruses by ethanol without addition of organic matter

Note: LRV, log10 reduction value
